# New strategy for virus discovery: viruses identified in human feces in the last decade

**DOI:** 10.1007/s11427-013-4516-y

**Published:** 2013-08-07

**Authors:** GuangCheng Xie, JieMei Yu, ZhaoJun Duan

**Affiliations:** National Institute of Viral Disease Control and Prevention, Chinese Center for Disease Control and Prevention, Beijing, 102206 China

**Keywords:** next-generation sequencing, novel viruses, metagenomic Koch’s postulates, viral metagenomics

## Abstract

Emerging and re-emerging viruses continue to surface all over the world. Some of these viruses have the potential for rapid and global spread with high morbidity and mortality, such as the SARS coronavirus outbreak. It is extremely urgent and important to identify a novel virus near-instantaneously to develop an active preventive and/or control strategy. As a culture-independent approach, viral metagenomics has been widely used to investigate highly divergent and completely new viruses in humans, animals, and even environmental samples in the past decade. A new model of Koch’s postulates, named the metagenomic Koch’s postulates, has provided guidance for the study of the pathogenicity of novel viruses. This review explains the viral metagenomics strategy for virus discovery and describes viruses discovered in human feces in the past 10 years using this approach. This review also addresses issues related to the metagenomic Koch’s postulates and the challenges for virus discovery in the future.

In the past decade, several serious emerging viral infectious diseases have left deep impression on many people. The world witnessed the first pandemic of the new millennium in 2003. Instead of the influenza virus, severe acute respiratory syndrome coronavirus (SARS-CoV) caused the first serious and widespread zoonotic disease, having a huge global impact on health, travel, and the economy [1–5]. The great global impact of the SARS outbreak was in some ways intensified by the delay in identifying the causative agent of the disease. Within a few months, SARS-CoV caused approximately 916 deaths and affected around 30 countries. When the novel SARS-CoV was isolated by a joint effort of the World Health Organization (WHO) SARS Collaborative Network, six months had passed. Hence, identifying the causative agent of a new epidemic is one of the most important steps for active control and prevention of viral disease outbreaks.

With the development of novel modern molecular biology technology, especially sequence-independent single-primer amplification (SISPA) [6], next-generation sequencing (NGS) such as 454-pyrosequencing [7,8], and viral metagenomics [9–11], multiple viruses can be detected simultaneously, and novel and highly divergent viruses can be discovered and genetically characterized quickly. A novel phlebovirus of the Bunyaviridae family, known as severe fever with thrombocytopenia syndrome bunyavirus, was discovered using SISPA technology [12,13]. Human bocavirus was also identified using the above-mentioned methods in 2005 [14]. Recently, a novel coronavirus, HCoV-EMC, was isolated in Jeddah, Saudi Arabia on June 13, 2012 [15]. Subsequently, the complete genome of MERS-hCoV was determined using an unbiased virus discovery approach involving NGS techniques, which determined that it was closely related to bat coronavirus but was distant from SARS-CoV [16]. This novel coronavirus, MERS-hCoV, reminds us to pay attention to animal coronaviruses, which may be the cause of severe disease in humans, and to develop strategies to rapidly determine unknown viral agents [17]. In fact, only limited data are available regarding the diversity of viruses present in humans and animals. A great number of human and animal viruses are still unknown.

## 1 Traditional methods of virus discovery

Before the advent of modern molecular methods, traditional virus discovery included filtration, tissue culture, electron microscopy, serology, and vaccination; cell culture for virus propagation was the virus discovery gold standard. However, many viruses cannot be easily propagated in cell culture, thus limiting our understanding of virology. Two milestone findings solved these difficulties, which allowed those interested in understanding new viruses first to amplify and then to sequence viral nucleic acids, namely DNA amplification by polymerase chain reaction (PCR) [18] and DNA sequencing with chain-terminating inhibitors (Sanger sequencing or first-generation sequencing) [19]. The number of sequences deposited on GenBank between August 2009 and August 2010 has totaled 970 million and 43 million bases of viral and phage origin, respectively, representing an annual growth of 20%–24% [20]. After that, several serious emerging viruses, such as Hendravirus [21], Nipah virus [22], Menangle virus [23], Melaka virus [24], and Reston Ebola virus [25], were discovered in different countries. The trend in virology research has shown gradual substitution of the traditional virus discovery methods with novel modern molecular biology technology. Nevertheless, traditional methods to isolate, identify, and characterize viruses play complementary roles in the virus discovery effort.

## 2 Novel modern molecular techniques of virus discovery

There are two major types of molecular methods for virus discovery, including sequence-dependent and sequence-independent methods. Sequence-dependent methods require knowledge of the nucleic acid sequence. Such methods include consensus primer PCR and microarrays. Several viruses, such as human immunodeficiency virus [26], simian retroviruses [27–29], and hepatitis E virus [30], have been identified using consensus sequences of known viruses; however, these methods have little or no value for characterizing completely novel viruses. Microarrays use probes that can hybridize to known viral sequences and potentially novel viruses with sufficient sequence similarity [31]. Microarrays have been applied in the detection of swine respiratory viruses in clinical samples [32] and for dengue virus [33]. Unlike sequence-dependent methods, the sequence-independent approaches do not rely on any knowledge of the virus. SISPA circumvents the viral load limitation of suppression subtractive hybridization (SSH). By now, there are several variations to the original protocol created by Reyes et al. [6]. The main strategy of SISPA is to exploit the sensitivity and the specificity of PCR amplification using primers that bind oligonucleotide fragments ligated to any putative viral DNA material in the sample. SISPA has been modified to allow the detection of both DNA and RNA viruses after the removal of genomic and contaminating nucleic acids [34]. Human astrovirus [35] and parvoviruses 2 and 3 [36] were discovered using SISPA methods. Another sequence-independent and culture-independent approach, viral metagenomics, provides superior capability for detecting known and unknown viruses and will be described in detail below.

## 3 Viral metagenomics

The term 'metagenome' was proposed earlier by Handelsman in PubMed, in 1998, in relation to classifying unculturable bacteria from soil samples [37]. Metagenomics can be defined as the characterization of genetic information directly from samples. It is a culture- and sequence-independent approach that does not rely on the presence of any particular gene in all subject entities [7,10]. This approach was originally developed as a tool for 'functional and sequence-based analysis of collective microbial genomes contained in environmental samples' [38,39]. The first application of metagenomics to the field of virology was in the analysis of viral communities sampled at two near-shore marine locations in San Diego, California [40]. Since then, viruses in numerous environments, including freshwater, marine sediment, soil, and the human gut, were surveyed. More information on published environmental viral metagenomes, DNA viral families, and RNA viral families in environmental samples and organisms found using the viral metagenomic approach can be found in Rosario's review [9]. The availability of NGS has forced viral metagenomics to be evaluated by unbiased means at previously unforeseen resolution and is providing a wealth of new opportunities in two major areas: viral candidate pathogen discovery and viral ecology [7].

Currently, several commercially available high-throughput sequencing platforms exist that vary by way of their sequencing principle, sequencing speed, expense, and read length. Figure 1 shows a schematic flow diagram summarizing the viral metagenomic studies that used sequence-independent amplification and high-throughput sequencing for virus discovery in 2012. A metagenomic analysis essentially entails the following steps: sample preparation, sequence-independent amplification, high-throughput sequenc-

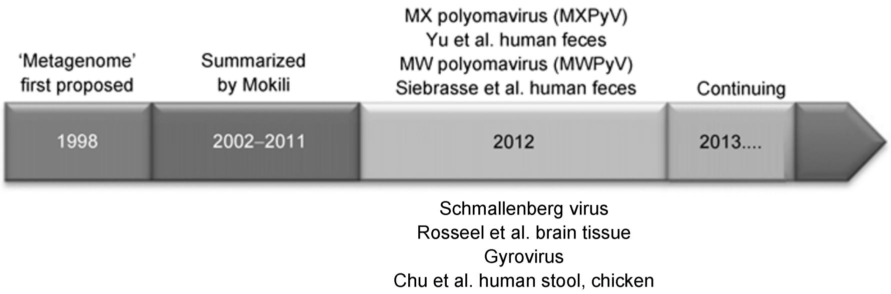

**Figure 1** Overview of viral metagenomic studies in 2012. Viruses discovered through viral metagenomic studies from 2002 to 2011 have been described in detail by Mokili et al. [10].

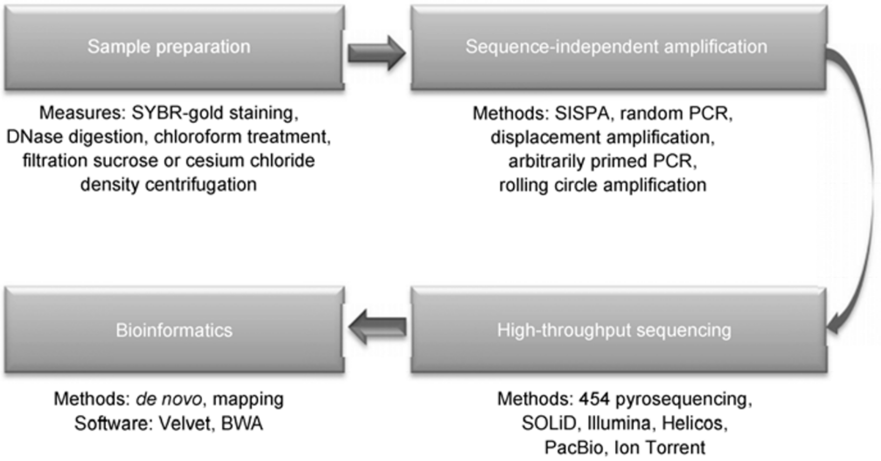

**Figure 2** Schematic flow diagram of a typical viral metagenomic study.

ing, and bioinformatics analysis (Figure 2). Below, we briefly discuss each step of this viral metagenomic process. More detailed descriptions can be obtained in a previously published paper [41].

(i) Sample preparation. Theoretically, any type of sample can be analyzed using the viral metagenomic approach; however, viral genomes are relatively short and of low concentration in many samples, and bacterial and eukaryotic nucleic acids can interfere with the isolation and detection of viral DNA or RNA. Thus, one of the most important, yet difficult, tasks of viral metagenomics is removal of non-viral nucleic acid while preserving viral nucleic acids [11,41,42]. Toward this aim, several measures have been taken, such as SYBR-gold staining, filtration, sucrose or cesium chloride density centrifugation [41], DNase digestion [43], and chloroform treatment [44]. Total RNA content is usually estimated by measuring ribosomal RNA (rRNA), because the abundance of rRNA and its association with ribosomes makes the detection of contaminating RNA viruses difficult. However, RNases are ineffective for degradation of rRNA. Therefore, other strategies must be employed. One rRNA depletion strategy is to use random primers during cDNA synthesis that do not target rRNA sequences [45,46]. Another strategy is to use biotin-labeled probes that target ribosomal sequences for depletion [47–49].

(ii) Sequence-independent amplification. Sequence-independent amplification is the important step in viral metagenomics that shows the true genetic composition of the sample and simultaneously amplifies several viral genomes and makes them available, including highly divergent and completely novel viruses, for characterization [11,34]. SISPA, random PCR amplification, displacement amplification, arbitrarily primed PCR, and rolling circle amplification are typically used for sequence-independent amplification. Detailed descriptions of these methods have been made available in previously published papers [11,50].

(iii) High-throughput sequencing. To identify known, highly divergent, or new viruses, sequencing is often utilized. The Sanger sequencing method can create high-quality sequence reads up to nearly 1000 nt; however, this highly laborious process limits its usage [43]. High-throughput sequencing, or NGS, is used more and more often in viral metagenomics. The 454 sequencing platform (Roche diagnostics) [8,51], SOLiD sequencing (Life Technologies) [7,52], Illumina sequencing [7,52], Helicos sequencing (Helicos Biosciences), PacBio sequencing (Pacific Biosciences), and Ion Torrent (Life Technologies) [7,52] are all commercial instruments in the marketplace, and all use slightly different methodologies to achieve clonal amplification and sequencing. The advantages and disadvantages of each of these instruments have been detailed in other papers [7,52–54].

(iv) Bioinformatics. Post-sequencing, the analysis of the vast amount of sequencing data produced, is the challenging part of viral metagenomics. The datasets from metagenomic studies are complicated, and they typically contain a mixture of different species. The genomes in the datasets are usually incomplete, with some cases having only a small number of short fragments belonging to each genome. There are two approaches that can be used for analysis of read data, *de novo* assembly using software such as Velvet [55] and mapping strategies using a mapper such as BWA [56]. Other programs and platforms also have been developed, the details of which can be found in the NGS review by Blomstrom et al. [50].

## 4 Viruses discovered in human feces

Feces comprise a community of many different microorganisms, including bacteria and viruses. Many viral diseases are transmitted by the oral-fecal route, and the viral agents are excreted in the feces. With the development of modern molecular methods and viral metagenomics, several new viruses have been discovered in feces in the past decade.

(i) Picornaviruses. Enteroviruses constitute the largest genus within the family Picornaviridae, which have non-enveloped, positive-sense, single-stranded RNA (+ssRNA) genomes surrounded by four structural proteins, VP1-VP4. A majority of these viruses infect humans and cause diseases ranging from minor respiratory illness to severe neurological disorders like meningitis, encephalitis, and poliomyelitis [57]. Metagenomic analyses of viruses in stool samples from children with acute flaccid paralysis (AFP) found numerous viruses, particularly HEV-C, including one from a potentially novel enterovirus genotype [58].

Human cosavirus (HCoSV) is a proposed new genus in the family Picornaviridae originally identified in 2008 in feces [59]. Since then, HCoSVs have been detected in feces from Australian [60] and Chinese [61] children. HCoSV infection with accompanying diarrhea in Thailand [62], the prevalence of a new species (F) of HCoSV, and another 26 new HCoSV genotypes in human feces of healthy children and children with AFP [63] were also reported. HCoSV pathogenicity in humans has remained unknown because detection rates in patients and healthy controls were similar in the only available cohort studies of patients with AFP in Asia [59] and with gastroenteritis in China [61]. In Brazil, the 3.6% detection rate in children with gastroenteritis [64] was comparable to the 1.8% rate in a cohort study of gastroenteritis in China [61]. The large number of HCoSV genotypes/serotypes reported indicates that HCoSV infection is associated with a range of diseases, such as unexplained diarrhea, AFP, or others. Future work will focus on defining whether HCoSV is a true human pathogen.

Two other novel picornaviruses have also been described since 2008 due to the advent of metagenomics: klassevirus [65] and salivirus [66]. The reported association between salivirus shedding and diarrhea indicates that such infections may account for a significant fraction of the unexplained cases of diarrhea occurring worldwide. Future studies to determine a possible link to disease in humans and any unique characteristics of the viral life cycle will be required. Viral culture in human cell lines, especially those from the gastrointestinal tract, could be suggestive that the virus is competent to replicate in human cells and that humans could be a bona fide host of klassevirus [65]. Further epidemiological screening and serological assays will be necessary to understand the diversity within this possible genus, the prevalence of these novel viruses, and the age range of those susceptible to infection.

(ii) Parvoviruses. Parvoviruses are, as their name suggests, small viruses, with a single-stranded DNA genome and are widespread pathogens that cause a wide range of diseases in humans and animals. Allander et al. [14] first reported the discovery of a previously unidentified human parvovirus in 2005. From 2009 to 2010, the bocavirus genus was expanded to include three additional species of human bocaviruses, HBoV2 [67], HBoV3 [68], and HBoV4 [69]. HBoV2-4 seems to be found primarily in human stool [67–71]. HBoV1 is predominantly a respiratory pathogen, whereas HBoV2 and possibly HBoV3 are associated with gastroenteritis [67,71]. A variety of signs and symptoms have been described in patients with HBoV infection, including rhinitis, pharyngitis, cough, dyspnea, and diarrhea, among others [72].

Human parvovirus 4 (PARV4) was identified in 2005 in a plasma sample [73]. Another two novel parvoviruses closely related to PARV4, porcine hokovirus and bovine hokovirus, were discovered in feces specimens from Hong Kong [74]. Furthermore, a proposed new Parvoviridae genus associated with acute diarrhea has been identified; however, wider geographic sampling of human and animal fecal samples will provide a better understanding of the genetic diversity of this proposed genus. Serological assays and case-control studies will also help determine whether members of this viral clade are associated with diarrhea or other symptoms [75].

(iii) Astroviruses. The family Astroviridae consists of small, non-lipid enveloped, single-stranded, positive-sense RNA viruses with genomes that contain three open reading frames (ORFs), designated ORF1a, ORF1b, and ORF2. Astroviruses are known to infect a variety of human and animal hosts. Recently, two highly divergent members of the astrovirus family, MLB1 [76–78] and VA1 [79], were identified and associated with human viral diarrhea. Astroviruses VA2, MLB2, and VA3 were also reported as novel astroviruses in human stool [80], as was HMOAstV A-C [81]. With the rising number of human astrovirus species detected in infections associated with unexplained AFP and/or diarrhea, increased understanding of the genetic diversity within viral families infecting humans will assist in future studies of their pathogenicity and allow the design of specific diagnostic assays [81].

(iv) Polyomaviruses. Viruses in the Polyomaviridae family typically possess ~5000 bp of circular, double-stranded DNA. The genome can be divided into three parts, including the regulatory region, the early region, and the later region. Over the past five years, seven novel polyomaviruses have been discovered in humans, namely KI polyomavirus (KIPyV) [82], WU polyomavirus (WUPyV) [83], Merkel cell polyomavirus (MCPyV) [84], human polyomavirus 6 (HPyV-6), human polyomavirus 7 (HPyV-7) [85], human polyomavirus 9 (HPyV-9) [86], and trichodysplasia spinulosa-associated polyomavirus (TSPyV) [87]. In 2012, another two novel polyomaviruses were discovered in human stool and provisionally named MW polyomavirus (MWPyV) [88] and MX polyomavirus (MXPyV) [89]. The number of polyomaviruses found in the human body continues to grow, raising the question of how many more species have yet to be identified and what roles they play in humans with and without manifest disease. Taking MWPyV as an example, one critical question is whether MWPyV is a bona fide infectious agent of humans and, if so, which disease(s), if any, might be associated with MWPyV infection. The detection of MWPyV in feces of children with diarrhea, many of which are unexplained, raises the possibility that MWPyV might play a role in human diarrhea. It is also possible that MWPyV is a dietary contaminant and does not actively infect humans. Serological studies and antibody-based immune responses to MWPyV and wider sampling and screening are approaches to determine whether MWPyV is a true infectious agent [88].

(v) Circoviruses. Members of the family Circoviridae are non-enveloped, spherical viruses with a single-stranded circular DNA genome of approximately 2 kb—the smallest known autonomously replicating viral genome [90]—and include the genera circovirus, gyrovirus, and gyclovirus (proposed) [91,92]. Until recently, chicken anemia virus (CAV) was the only known representative of the gyrovirus genus. In the last two years, three novel gyroviruses have been reported: avian gyrovirus 2 (AGV2) in chickens [93], a human gyrovirus (HGV) detected on human skin [94], and gyrovirus 3 (GyV3) in human feces and chicken meat [95]. Recently, a novel gyrovirus 4 (GyV4) was also identified in human stool and in chicken meat prepared for human consumption [96]. In addition, a new strain of CAV, designated GD-1-12, was isolated from fecal samples of a 12-day-old commercial broiler in Guangdong province, China [97]. Multiple diverse circoviruses were found in human and chimpanzee feces, such as CyCV-PK5006 and Chimp 17 [92]. Other novel single-stranded, circular DNA viruses also detected were found in porcine [98] and bovine feces [99], respectively.

Diarrhea is the third leading infectious cause of death worldwide, and an estimated 1.4 billion nonfatal episodes occur yearly [100,101]. Importantly, it is estimated that 40% of diarrhea cases are of unknown etiology [60,102]. Viral metagenomics has largely propelled the progress in virus discovery; however, the association of these newly-discovered viruses with specific diseases is largely unknown. An understanding of these newly-discovered viruses' disease associations and causations is extremely urgent.

## 5 Metagenomic Koch's postulates

Viral metagenomics has provided a powerful tool for discovering new viruses. However, virus discovery is only the first step to determining the etiology behind a disease. The detection of nucleic acid is not sufficient to prove causality. For that, more comprehensive studies have to be performed [103]. Evidence for causality is not always conclusive, even when the suspect virus is found "at the scene of the crime". This means that finding a virus in a sample from a patient with an illness of unknown etiology, and even demonstrating an association, does not always prove causation. Therefore, strict guidelines proposed by Robert Koch and later modified by Rivers [104] have been used to assign causality to infectious agents [10]. One of Koch's postulates requires that the candidate etiological agent be isolated from a diseased organism and grown in pure culture. However, not all viruses can be propagated by current culture methods [103].

In many cases, Koch's postulates will not be satisfied if current culture standards are used to prove causality. Koch's postulates have since been modified to acknowledge molecular methods used to monitor the role played by genes in bacterial virulence [105]. The revised so-called molecular Koch's postulates can be applied to pathogenic members of a genus or pathogenic strains of a species as well as non-pathogenic strains. Because genes can be expressed at different time points during infection, however, new molecular methods do not always distinctively characterize virulence genes and make a clear association with the disease of unknown etiology. Mokili et al. [10] proposed the so-called metagenomic Koch's postulates, which focus on the identification of metagenomic traits in disease subjects (Figure 3). Molecular markers such as sequence reads, assembled contigs, genes, or full genomes that can uniquely distinguish disease-associated metagenomes from those obtained from matched healthy control subjects can all be used to define metagenomic traits. Thus, satisfying metagenomic Koch's

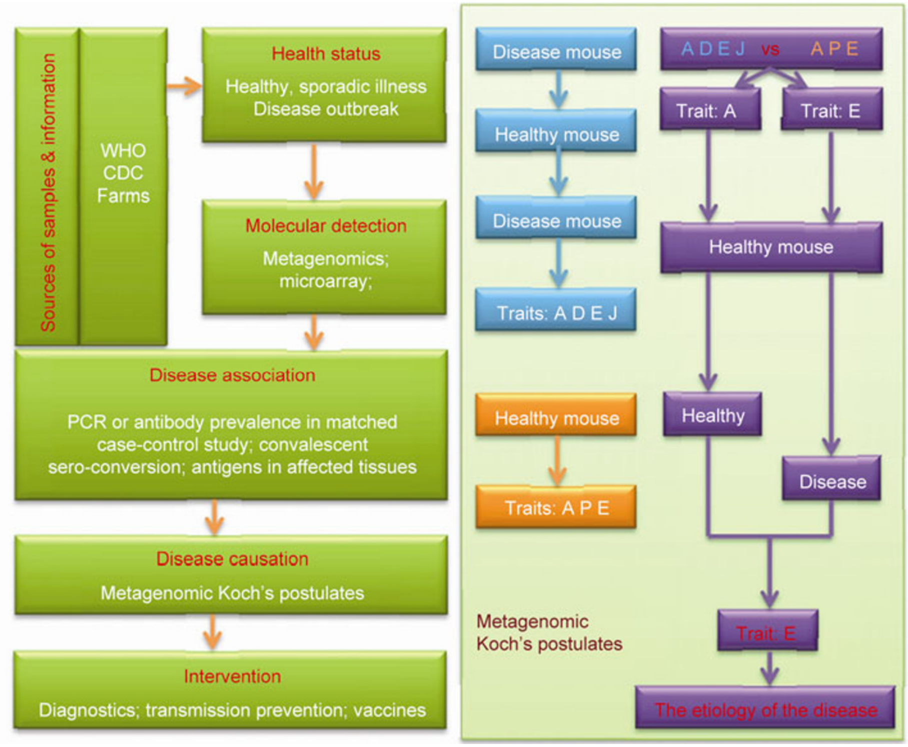

**Figure 3** Flow chart of virus discovery, pathogenicity determination, intervention, and metagenomic Koch's postulates. Traits A, D, E, and J found in the diseased animal (e.g., a mouse) are not present in the healthy control (represented in blue). The acquisition or increase of new metagenomic traits, A, P, and E, are represented in orange. Inoculation of the suspected purified traits into a healthy mouse will induce disease if the traits encompass the etiology of the disease (represented in purple); pathogenicity as a trait is represented in red. The figure is modified from models proposed by Mokili et al. [10] and Delwart [106].

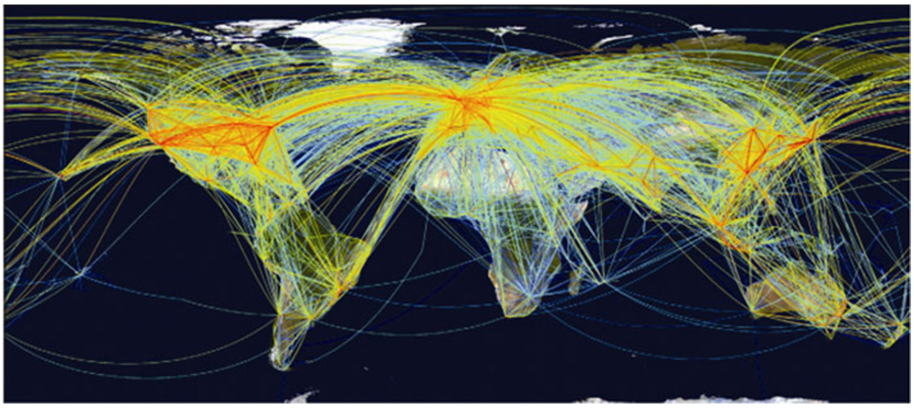

**Figure 4** The global aviation network. Lines show direct links between airports, and the color of the line indicates passenger capacity in people per day (thousands (red), hundreds (yellow), and tens (blue)) [110].

postulates is possible when one or multiple viral agents are involved in disease causation.

## 6 Challenges in virus discovery

Novel viruses are still emerging. In November 2011, a novel orthobunyavirus named Schmallenberg virus (SBV) was detected in plasma samples from a farm near the German town of Schmallenberg by using a metagenomic approach with NGS [107]; this novel virus is on the rise in Europe [108,109]. The SBV epidemic showed once again that the novel technology of metagenomics is very useful for early detection of novel pathogens in livestock. In fact, veterinary diagnostics in Europe has proved to be a very effective network of institutions studying epizootic diseases. Before any strategy is decided for prediction, precaution, and prevention of EIDs in humans, three questions should always be asked and answered: what to expect, what to be prepared for, and what to do.

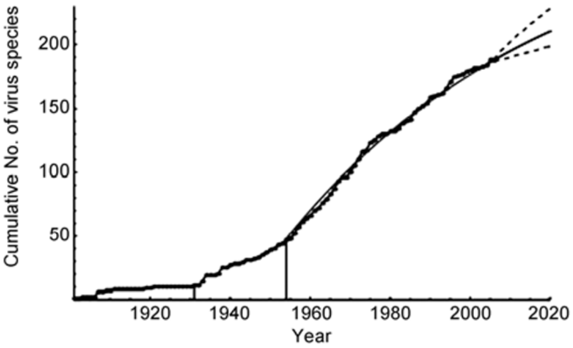

**Figure 5** The discovery curve for human virus species. The cumulative number of species reported to infect humans (black circles and line). Statistically significant upward breakpoints are shown (vertical lines). The best-fit curve (solid line) and lower and upper 95% posterior intervals (dashed lines) for extrapolation to 2020 are shown [115].

Another important issue is that of emerging vector-borne diseases in global health. Many vector-borne pathogens have appeared in new regions in the past two decades. Anthropogenic trade, travel, as well as hosts, vectors, and climate conditions all contribute to emerging vector-borne disease. Figure 4 shows the direct links between airports spanning across the world. Knowing the drivers, dynamics, and ecology of zoonoses are all helpful for prediction and prevention of the next pandemic zoonosis [110–112].

The Virus Pathogen Database and Analysis Resource (ViPR; www.ViPRbrc.org) is an integrated repository of data and analysis tools for multiple virus families and is supported by the National Institute of Allergy and Infectious Diseases (NIAID) Bioinformatics Resource Centers (BRC) program. This program has the advantage of a powerful suite of resources provided by the ViPR BRC with which virology researchers can streamline and expedite experimental discovery for the ultimate goal of developing improved diagnostics, prophylactics, and/or therapeutics for pathogenic viruses [113,114]. With globally coordinated activity and effort, temporal trends in the discovery of human viruses [115] (Figure 5) will enable the control of forthcoming pandemic zoonoses and lead to greater achievements.

1 Fouchier R A, Kuiken T, Schutten M, et al. Aetiology: Koch's postulates fulfilled for SARS virus. Nature, 2003, 423: 240

2 Drosten C, Gunther S, Preiser W, et al. Identification of a novel coronavirus in patients with severe acute respiratory syndrome. N Engl J Med, 2003, 348: 1967–1976

3 Marra M A, Jones S J, Astell C R, et al. The Genome sequence of the SARS-associated coronavirus. Science, 2003, 300: 1399–1404

4 Peiris J S, Guan Y, Yuen K Y. Severe acute respiratory syndrome. Nat Med, 2004, 10: S88–S97

5 Rota P A, Oberste M S, Monroe S S, et al. Characterization of a novel coronavirus associated with severe acute respiratory syndrome. Science, 2003, 300: 1394–1399

6 Reyes G R, Kim J P. Sequence-independent, single-primer amplification (SISPA) of complex DNA populations. Mol Cell Probes, 1991, 5: 473–481

7 Radford A D, Chapman D, Dixon L, et al. Application of next-generation sequencing technologies in virology. J Gen Virol, 2012, 93: 1853–1868

8 Margulies M, Egholm M, Altman W E, et al. Genome sequencing in microfabricated high-density picolitre reactors. Nature, 2005, 437: 376–380

9 Rosario K, Breitbart M. Exploring the viral world through metagenomics. Curr Opin Virol, 2011, 1: 289–297

10 Mokili J L, Rohwer F, Dutilh B E. Metagenomics and future perspectives in virus discovery. Curr Opin Virol, 2012, 2: 63–77

11 Delwart E L. Viral metagenomics. Rev Med Virol, 2007, 17: 115–131

12 Yu X J, Liang M F, Zhang S Y, et al. Fever with thrombocytopenia associated with a novel bunyavirus in China. N Engl J Med, 2011, 364: 1523–1532

13 Wu Y, Gao G F. Severe fever with thrombocytopenia syndrome virus expands its borders. Emerg Microbes Infect, 2013, in press

14 Allander T, Tammi M T, Eriksson M, et al. Cloning of a human parvovirus by molecular screening of respiratory tract samples. Proc Natl Acad Sci USA, 2005, 102: 12891–12896

15 Zaki A M, van Boheemen S, Bestebroer T M, et al. Isolation of a novel coronavirus from a man with pneumonia in Saudi Arabia. N Engl J Med, 2012, 367: 1814–1820

16 van Boheemen S, de Graaf M, Lauber C, et al. Genomic characterization of a newly discovered coronavirus associated with acute respiratory distress syndrome in humans. MBio, 2012, 3: e00473–12

17 Chan J F, Li K S, To K K, et al. Is the discovery of the novel human betacoronavirus 2c EMC/2012 (HCoV-EMC) the beginning of another SARS-like pandemic? J Infect, 2012, 65: 477–489

18 Mullis K, Faloona F, Scharf S, et al. Specific enzymatic amplification of DNA *in vitro*: the polymerase chain reaction. Cold Spring Harb Symp Quant Biol, 1986, 51(Pt 1): 263–273

19 Sanger F, Nicklen S, Coulson A R. DNA sequencing with chain-terminating inhibitors. Proc Natl Acad Sci USA, 1977, 74: 5463–5467

20 Benson D A, Karsch-Mizrachi I, Lipman D J, et al. GenBank. Nucleic Acids Res, 2011, 39: D32–D37

21 Murray K, Selleck P, Hooper P, et al. A morbillivirus that caused fatal disease in horses and humans. Science, 1995, 268: 94–97

22 Chua K B, Bellini W J, Rota P A, et al. Nipah virus: a recently emergent deadly paramyxovirus. Science, 2000, 288: 1432–1435

23 Philbey A W, Kirkland P D, Ross A D, et al. An apparently new virus (family Paramyxoviridae) infectious for pigs, humans, and fruit bats. Emerg Infect Dis, 1998, 4: 269–271

24 Chua K B, Crameri G, Hyatt A, et al. A previously unknown reovirus of bat origin is associated with an acute respiratory disease in humans. Proc Natl Acad Sci USA, 2007, 104: 11424–11429

25 Barrette R W, Metwally S A, Rowland J M, et al. Discovery of swine as a host for the Reston ebolavirus. Science, 2009, 325: 204–206

26 Mokili J L, Rogers M, Carr J K, et al. Identification of a novel clade of human immunodeficiency virus type 1 in Democratic Republic of Congo. AIDS Res Hum Retroviruses, 2002, 18: 817–823

27 Takemura T, Ekwalanga M, Bikandou B, et al. A novel simian immunodeficiency virus from black mangabey (*Lophocebus aterrimus*) in the Democratic Republic of Congo. J Gen Virol, 2005, 86: 1967–1971

28 Barlow K L, Ajao A O, Clewley J P. Characterization of a novel simian immunodeficiency virus (SIVmonNG1) genome sequence from a mona monkey (Cercopithecus mona). J Virol, 2003, 77: 6879–6888

29 Clewley J P, Lewis J C, Brown D W, et al. A novel simian immunodeficiency virus (SIVdrl) pol sequence from the drill monkey, *Mandrillus leucophaeus*. J Virol, 1998, 72: 10305–10309

30 Reyes G R, Purdy M A, Kim J P, et al. Isolation of a cDNA from the virus responsible for enterically transmitted non-A, non-B hepatitis. Science, 1990, 247: 1335–1339

31 Chou C C, Lee T T, Chen C H, et al. Design of microarray probes for virus identification and detection of emerging viruses at the genus level. BMC Bioinformatics, 2006, 7: 232

32 Nicholson T L, Kukielka D, Vincent A L, et al. Utility of a panviral microarray for detection of swine respiratory viruses in clinical samples. J Clin Microbiol, 2011, 49: 1542–1548

33 Yozwiak N L, Skewes-Cox P, Stenglein M D, et al. Virus identification in unknown tropical febrile illness cases using deep sequencing. PLoS Negl Trop Dis, 2012, 6: e1485

34 Bexfield N, Kellam P. Metagenomics and the molecular identification of novel viruses. Vet J, 2011, 190: 191–198

35 Finkbeiner S R, Li Y, Ruone S, et al. Identification of a novel astrovirus (astrovirus VA1) associated with an outbreak of acute gastroenteritis. J Virol, 2009, 83: 10836–10839

36 Allander T, Emerson S U, Engle R E, et al. A virus discovery method incorporating DNase treatment and its application to the identification of two bovine parvovirus species. Proc Natl Acad Sci USA, 2001, 98: 11609–11614

37 Handelsman J, Rondon M R, Brady S F, et al. Molecular biological access to the chemistry of unknown soil microbes: a new frontier for natural products. Chem Biol, 1998, 5: R245–R249

38 Riesenfeld C S, Schloss P D, Handelsman J. Metagenomics: genomic analysis of microbial communities. Annu Rev Genet, 2004, 38: 525–552

39 Schloss P D, Handelsman J. Biotechnological prospects from metagenomics. Curr Opin Biotechnol, 2003, 14: 303–310

40 Breitbart M, Salamon P, Andresen B, et al. Genomic analysis of uncultured marine viral communities. Proc Natl Acad Sci USA, 2002, 99: 14250–14255

41 Thurber R V, Haynes M, Breitbart M, et al. Laboratory procedures to generate viral metagenomes. Nat Protoc, 2009, 4: 470–483

42 Edwards R A, Rodriguez-Brito B, Wegley L, et al. Using pyrosequencing to shed light on deep mine microbial ecology. BMC Genomics, 2006, 7: 57

43 Breitbart M, Rohwer F. Method for discovering novel DNA viruses in blood using viral particle selection and shotgun sequencing. Biotechniques, 2005, 39: 729–736

44 Willner D, Furlan M, Schmieder R, et al. Metagenomic detection of phage-encoded platelet-binding factors in the human oral cavity. Proc Natl Acad Sci USA, 2011, 108(Suppl 1): 4547–4553

45 Endoh D, Mizutani T, Kirisawa R, et al. Species-independent detection of RNA virus by representational difference analysis using non-ribosomal hexanucleotides for reverse transcription. Nucleic Acids Res, 2005, 33: e65

46 Armour C D, Castle J C, Chen R, et al. Digital transcriptome profiling using selective hexamer priming for cDNA synthesis. Nat Methods, 2009, 6: 647–649

47 He S, Wurtzel O, Singh K, et al. Validation of two ribosomal RNA removal methods for microbial metatranscriptomics. Nat Methods, 2010, 7: 807–812

48 Stewart F J, Ottesen E A, DeLong E F. Development and quantitative analyses of a universal rRNA-subtraction protocol for microbial metatranscriptomics. ISME J, 2010, 4: 896–907

49 Chen Z, Duan X. Ribosomal RNA depletion for massively parallel bacterial RNA-sequencing applications. Methods Mol Biol, 2011, 733: 93–103

50 Blomstrom A L. Viral metagenomics as an emerging and powerful tool in veterinary medicine. Vet Q, 2011, 31: 107–114

51 Gilles A, Meglecz E, Pech N, et al. Accuracy and quality assessment of 454 GS-FLX Titanium pyrosequencing. BMC Genomics, 2011, 12: 245

52 Loman N J, Misra R V, Dallman T J, et al. Performance comparison of benchtop high-throughput sequencing platforms. Nat Biotechnol, 2012, 30: 434–439

53 Quail M A, Smith M, Coupland P, et al. A tale of three next generation sequencing platforms: comparison of Ion Torrent, Pacific Biosciences and Illumina MiSeq sequencers. BMC Genomics, 2012, 13: 341

54 Liu L, Li Y, Li S, et al. Comparison of next-generation sequencing systems. J Biomed Biotechnol, 2012, 2012: 251364

55 Zerbino D R, Birney E. Velvet: algorithms for de novo short read assembly using de Bruijn graphs. Genome Res, 2008, 18: 821–829

56 Li H, Durbin R. Fast and accurate short read alignment with Burrows-Wheeler transform. Bioinformatics, 2009, 25: 1754–1760

57 Berlin L E, Rorabaugh M L, Heldrich F, et al. Aseptic meningitis in infants <2 years of age: diagnosis and etiology. J Infect Dis, 1993, 168: 888–892

58 Victoria J G, Kapoor A, Li L, et al. Metagenomic analyses of viruses in stool samples from children with acute flaccid paralysis. J Virol, 2009, 83: 4642–4651

59 Kapoor A, Victoria J, Simmonds P, et al. A highly prevalent and genetically diversified Picornaviridae genus in South Asian children. Proc Natl Acad Sci USA, 2008, 105: 20482–20487

60 Holtz L R, Finkbeiner S R, Kirkwood C D, et al. Identification of a novel picornavirus related to cosaviruses in a child with acute diarrhea. Virol J, 2008, 5: 159

61 Dai X Q, Hua X G, Shan T L, et al. Human cosavirus infections in children in China. J Clin Virol, 2010, 48: 228–229

62 Khamrin P, Chaimongkol N, Malasao R, et al. Detection and molecular characterization of cosavirus in adults with diarrhea, Thailand. Virus Genes, 2012, 44: 244–246

63 Kapusinszky B, Phan T G, Kapoor A, et al. Genetic diversity of the genus *Cosavirus* in the family Picornaviridae: a new species, recombination, and 26 new genotypes. PLoS ONE, 2012, 7: e36685

64 Stocker A, Souza B F, Ribeiro T C, et al. Cosavirus infection in persons with and without gastroenteritis, Brazil. Emerg Infect Dis, 2012, 18: 656–659

65 Greninger A L, Runckel C, Chiu C Y, et al. The complete genome of klassevirus—a novel picornavirus in pediatric stool. Virol J, 2009, 6: 82

66 Li L, Victoria J, Kapoor A, et al. A novel picornavirus associated with gastroenteritis. J Virol, 2009, 83: 12002–12006

67 Arthur J L, Higgins G D, Davidson G P, et al. A novel bocavirus associated with acute gastroenteritis in Australian children. PLoS Pathog, 2009, 5: e1000391

68 Kapoor A, Slikas E, Simmonds P, et al. A newly identified bocavirus species in human stool. J Infect Dis, 2009, 199: 196–200

69 Kapoor A, Simmonds P, Slikas E, et al. Human bocaviruses are highly diverse, dispersed, recombination prone, and prevalent in enteric infections. J Infect Dis, 2010, 201: 1633–1643

70 Chieochansin T, Kapoor A, Delwart E, et al. Absence of detectable replication of human bocavirus species 2 in respiratory tract. Emerg Infect Dis, 2009, 15: 1503–1505

71 Chow B D, Ou Z, Esper F P. Newly recognized bocaviruses (HBoV, HBoV2) in children and adults with gastrointestinal illness in the United States. J Clin Virol, 2010, 47: 143–147

72 Jartti T, Hedman K, Jartti L, et al. Human bocavirus—the first 5 years. Rev Med Virol, 2012, 22: 46–64

73 Jones M S, Kapoor A, Lukashov V V, et al. New DNA viruses identified in patients with acute viral infection syndrome. J Virol, 2005, 79: 8230–8236

74 Lau S K, Woo P C, Tse H, et al. Identification of novel porcine and bovine parvoviruses closely related to human parvovirus 4. J Gen Virol, 2008, 89: 1840–1848

75 Phan T G, Vo N P, Bonkoungou I J, et al. Acute diarrhea in West African children: diverse enteric viruses and a novel parvovirus genus. J Virol, 2012, 86: 11024–11030

76 Finkbeiner S R, Allred A F, Tarr P I, et al. Metagenomic analysis of human diarrhea: viral detection and discovery. PLoS Pathog, 2008, 4: e1000011

77 Finkbeiner S R, Kirkwood C D, Wang D. Complete genome sequence of a highly divergent astrovirus isolated from a child with acute diarrhea. Virol J, 2008, 5: 117

78 Finkbeiner S R, Le B M, Holtz L R, et al. Detection of newly described astrovirus MLB1 in stool samples from children. Emerg Infect Dis, 2009, 15: 441–444

79 Finkbeiner S R, Li Y, Ruone S, et al. Identification of a novel astrovirus (astrovirus VA1) associated with an outbreak of acute gastroenteritis. J Virol, 2009, 83: 10836–10839

80 Finkbeiner S R, Holtz L R, Jiang Y, et al. Human stool contains a previously unrecognized diversity of novel astroviruses. Virol J, 2009, 6: 161
81 Kapoor A, Li L, Victoria J, et al. Multiple novel astrovirus species in human stool. J Gen Virol, 2009, 90: 2965–2972
82 Allander T, Andreasson K, Gupta S, et al. Identification of a third human polyomavirus. J Virol, 2007, 81: 4130–4136
83 Gaynor A M, Nissen M D, Whiley D M, et al. Identification of a novel polyomavirus from patients with acute respiratory tract infections. PLoS Pathog, 2007, 3: e64
84 Feng H, Shuda M, Chang Y, et al. Clonal integration of a polyomavirus in human Merkel cell carcinoma. Science, 2008, 319: 1096–1100
85 Schowalter R M, Pastrana D V, Pumphrey K A, et al. Merkel cell polyomavirus and two previously unknown polyomaviruses are chronically shed from human skin. Cell Host Microbe, 2010, 7: 509–515
86 Scuda N, Hofmann J, Calvignac-Spencer S, et al. A novel human polyomavirus closely related to the african green monkey-derived lymphotropic polyomavirus. J Virol, 2011, 85: 4586–4590
87 van der Meijden E, Janssens R W, Lauber C, et al. Discovery of a new human polyomavirus associated with trichodysplasia spinulosa in an immunocompromized patient. PLoS Pathog, 2010, 6: e1001024
88 Siebrasse E A, Reyes A, Lim E S, et al. Identification of MW polyomavirus, a novel polyomavirus in human stool. J Virol, 2012, 86: 10321–10326
89 Yu G, Greninger A L, Isa P, et al. Discovery of a novel polyomavirus in acute diarrheal samples from children. PLoS ONE, 2012, 7: e49449
90 Fringuelli E, Scott A N, Beckett A, et al. Diagnosis of duck circovirus infections by conventional and real-time polymerase chain reaction tests. Avian Pathol, 2005, 34: 495–500
91 Blinkova O, Victoria J, Li Y, et al. Novel circular DNA viruses in stool samples of wild-living chimpanzees. J Gen Virol, 2010, 91: 74–86
92 Li L, Kapoor A, Slikas B, et al. Multiple diverse circoviruses infect farm animals and are commonly found in human and chimpanzee feces. J Virol, 2010, 84: 1674–1682
93 Rijsewijk F A, Dos S H, Teixeira T F, et al. Discovery of a genome of a distant relative of chicken anemia virus reveals a new member of the genus Gyrovirus. Arch Virol, 2011, 156: 1097–1100
94 Sauvage V, Cheval J, Foulongne V, et al. Identification of the first human gyrovirus, a virus related to chicken anemia virus. J Virol, 2011, 85: 7948–7950
95 Phan T G, Li L, O'Ryan M G, et al. A third gyrovirus species in human faeces. J Gen Virol, 2012, 93: 1356–1361
96 Chu D K, Poon L L, Chiu S S, et al. Characterization of a novel gyrovirus in human stool and chicken meat. J Clin Virol, 2012, 55: 209–213
97 Zhang X, Xie Q, Ji J, et al. Complete genome sequence analysis of a recent chicken anemia virus isolate and comparison with a chicken anemia virus isolate from human fecal samples in China. J Virol, 2012, 86: 10896–10897
98 Sikorski A, Arguello-Astorga G R, Dayaram A, et al. Discovery of a novel circular single-stranded DNA virus from porcine faeces. Arch Virol, 2013, 158: 283–289
99 Kim H K, Park S J, Nguyen V G, et al. Identification of a novel single-stranded, circular DNA virus from bovine stool. J Gen Virol, 2012, 93: 635–639
100 Kosek M, Bern C, Guerrant R L. The global burden of diarrhoeal disease, as estimated from studies published between 1992 and 2000. Bull World Health Organ, 2003, 81: 197–204
101 O''Ryan M, Prado V, Pickering L K. A millennium update on pediatric diarrheal illness in the developing world. Semin Pediatr Infect Dis, 2005, 16: 125–136
102 Denno D M, Stapp J R, Boster D R, et al. Etiology of diarrhea in pediatric outpatient settings. Pediatr Infect Dis J, 2005, 24: 142–148
103 Fredericks D N, Relman D A. Sequence-based identification of microbial pathogens: a reconsideration of Koch's postulates. Clin Microbiol Rev, 1996, 9: 18–33
104 Rivers T M. Viruses and Koch's Postulates. J Bacteriol, 1937, 33: 1–12
105 Falkow S. Molecular Koch's postulates applied to microbial pathogenicity. Rev Infect Dis, 1988, 10(Suppl 2): S274–S276
106 Delwart E. Animal virus discovery: improving animal health, understanding zoonoses, and opportunities for vaccine development. Curr Opin Virol, 2012, 2: 344–352
107 Hoffmann B, Scheuch M, Hoper D, et al. Novel orthobunyavirus in Cattle, Europe, 2011. Emerg Infect Dis, 2012, 18: 469–472
108 Garigliany M M, Bayrou C, Kleijnen D, et al. Schmallenberg virus: a new Shamonda/Sathuperi-like virus on the rise in Europe. Antiviral Res, 2012, 95: 82–87
109 Beer M, Conraths F J, van der Poel W H. 'Schmallenberg virus'—a novel orthobunyavirus emerging in Europe. Epidemiol Infect, 2013, 141: 1–8
110 Kilpatrick A M, Randolph S E. Drivers, dynamics, and control of emerging vector-borne zoonotic diseases. Lancet, 2012, 380: 1946–1955
111 Morse S S, Mazet J A, Woolhouse M, et al. Prediction and prevention of the next pandemic zoonosis. Lancet, 2012, 380: 1956–1965
112 Karesh W B, Dobson A, Lloyd-Smith J O, et al. Ecology of zoonoses: natural and unnatural histories. Lancet, 2012, 380: 1936–1945
113 Pickett B E, Greer D S, Zhang Y, et al. Virus pathogen database and analysis resource (ViPR): a comprehensive bioinformatics database and analysis resource for the coronavirus research community. Viruses, 2012, 4: 3209–3226
114 Pickett B E, Sadat E L, Zhang Y, et al. ViPR: an open bioinformatics database and analysis resource for virology research. Nucleic Acids Res, 2012, 40: D593–D598
115 Woolhouse M E, Howey R, Gaunt E, et al. Temporal trends in the discovery of human viruses. Proc Biol Sci, 2008, 275: 2111–2115

**Open Access** This article is distributed under the terms of the Creative Commons Attribution License which permits any use, distribution, and reproduction in any medium, provided the original author(s) and source are credited.
